# Sex-specific outcomes following chorioamnionitis

**DOI:** 10.1038/s41390-026-05336-2

**Published:** 2026-07-28

**Authors:** Jessica E. Knobbe, Jennifer R. Bermick

**Affiliations:** 1 Stead Family Depsartment of Pediatrics, The University of Iowa Roy J and Lucille A Carver College of Medicine, Iowa City, IA, USA.; 2 Interdisciplinary Graduate Program in Immunology, Medical Scientist Training Program, The University of Iowa Roy J and Lucille A Carver College of Medicine, Iowa City, IA, USA.

## Abstract

Chorioamnionitis is a common cause of perinatal inflammation and is associated with adverse neonatal and long-term outcomes. Although male infants experience higher rates and greater severity of many inflammation-related morbidities, the contribution of fetal sex to chorioamnionitis-related outcomes remains understudied. This review synthesizes evidence from human studies and animal models to evaluate how sexually dimorphic placental biology and fetal immune responses shape offspring vulnerability to inflammation-induced injury. Across species, males consistently exhibit heightened acute inflammatory responses, increased neuroinflammation, and greater structural brain vulnerability following exposure to perinatal inflammation. Human studies demonstrate sex-specific alterations in cerebral blood flow and brain metabolites, with males showing patterns that place them at increased risk of cerebral palsy and neurodevelopmental impairment. Females, while mounting distinct inflammatory responses, show susceptibility in other organ systems, including impaired lung function and altered metabolic trajectories in childhood. Findings from animal models parallel human observations and reveal possible mechanistic pathways through which sex modifies neuroimmune, metabolic, and behavioral outcomes across the lifespan. Collectively, this review highlights the substantial impact of fetal sex on inflammation-driven morbidity and underscores the critical need to incorporate sex as a biological variable in perinatal research and clinical risk stratification.

## INTRODUCTION

**Fig. 1:**
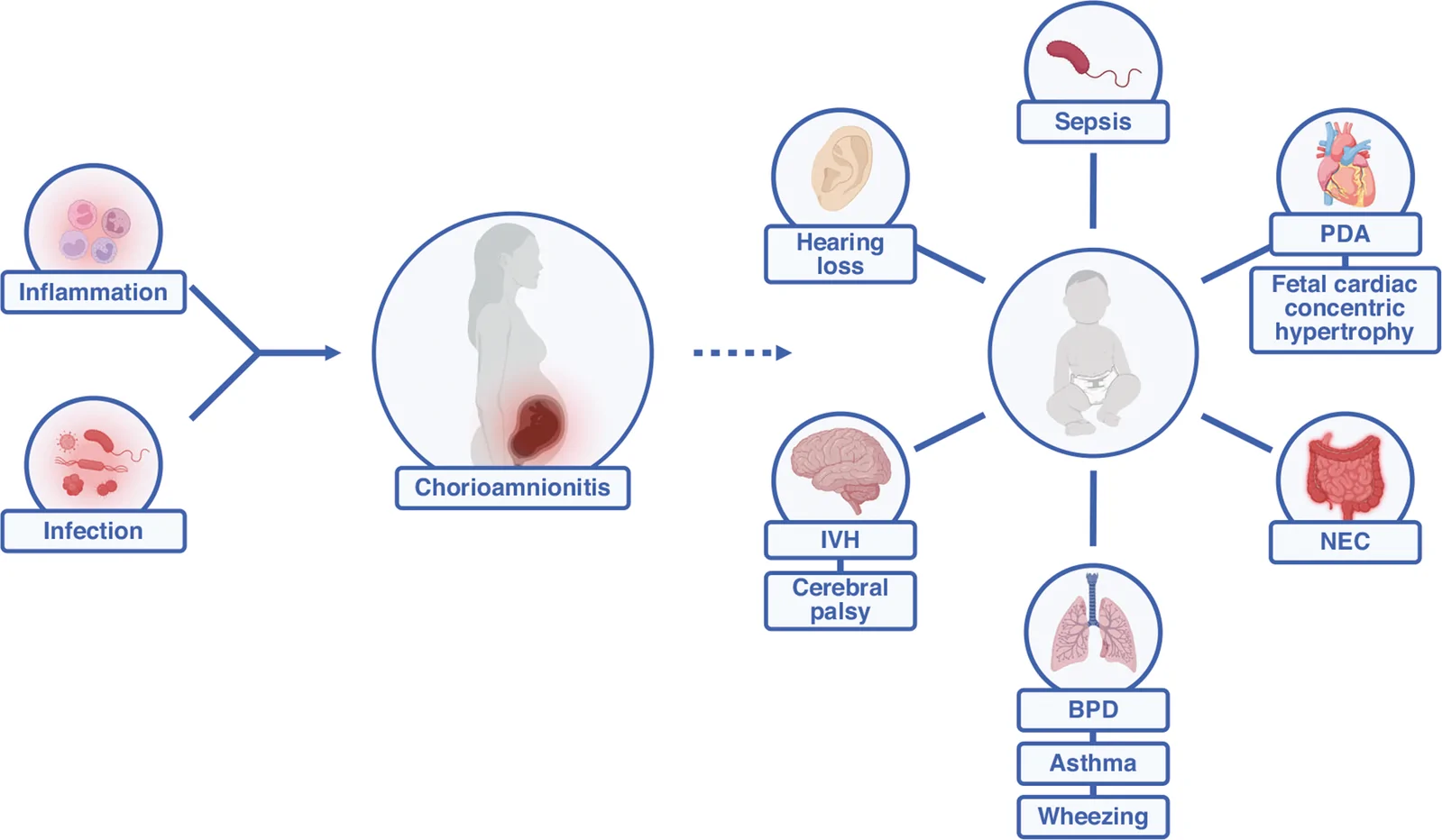
Chorioamnionitis is an independent risk factor for several neonatal morbidities. Chorioamnionitis affects many organs and increases the risk of several morbidities, including both early- and late-onset sepsis, patent ductus arteriosus (PDA), fetal cardiac concentric hypertrophy, necrotizing enterocolitis (NEC), bronchopulmonary dysplasia (BPD), asthma, wheezing, intraventricular hemorrhage (IVH), cerebral palsy, and hearing loss. Figure made using BioRender.

Chorioamnionitis (or “Triple I” in abbreviation of “intrauterine inflammation or infection”^1^) is acute infection and/or inflammation of the fetal membranes and the placenta. Chorioamnionitis is a common condition associated with pregnancy and is reported to complicate 40–70% of premature births^2^ and 1–10% of term births.^2–4^ Chorioamnionitis is diagnosed clinically by maternal fever in combination with fetal tachycardia (> 160 beats per minute for ≥10 min), maternal leukocytosis (white blood cell count >15,000/mm^3^), or purulent fluid from the cervical os.^1^ The presence of histological chorioamnionitis can be confirmed by leukocytic infiltration into the chorioamniotic membranes on review of the placenta by a pathologist.^5,6^ This infiltrate is primarily neutrophilic,^6^ and results in a robust inflammatory response by both the mother^7^ and the fetus.^7,8^ Following birth, the chorioamnionitis-exposed neonate is at higher risk of developing a variety of morbidities, including both early- and late-onset sepsis, patent ductus arteriosus, necrotizing enterocolitis, bronchopulmonary dysplasia, cerebral palsy, and intraventricular hemorrhage (Fig. 1).^9,10^ Many studies demonstrate that chorioamnionitis is a risk factor for these morbidities independent of prematurity.^11–17^

**Fig. 2:**
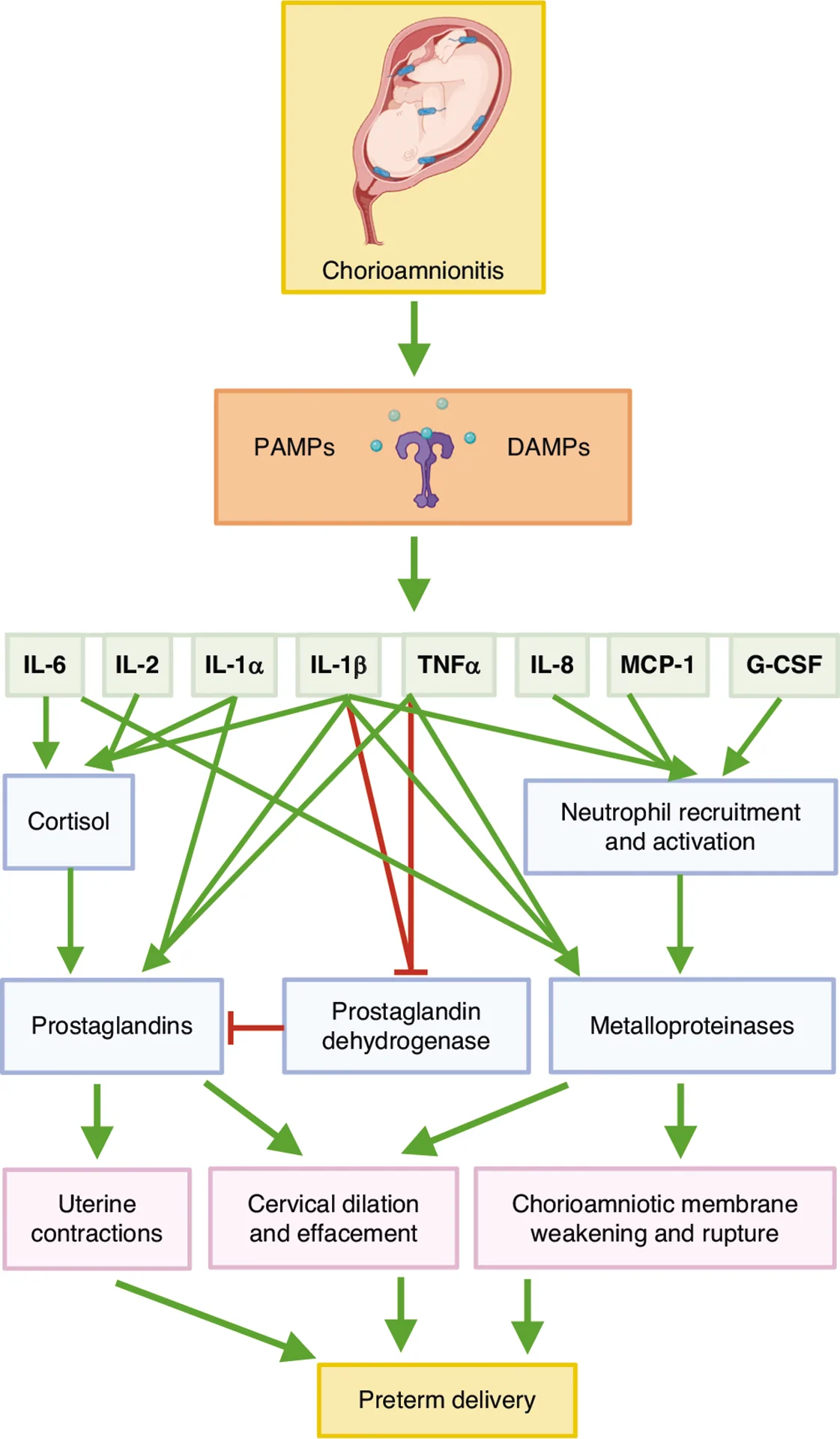
Chorioamnionitis induces preterm birth through upregulation of inflammatory cytokines. The most common cause of chorioamnionitis is thought to be ascending bacterial infection. However, chorioamnionitis may be caused by other infectious agents or may be due to sterile inflammation. Pattern recognition receptors (PRRs), such as toll-like receptors (TLRs), identify pathogen-associated or damage-associated molecular patterns (PAMPs and DAMPs). TLRs and other PRRs are expressed by both maternal-derived and fetal-derived placental cells. During chorioamnionitis, these cells may respond to PAMPs and/or DAMPs by production of a variety of different inflammatory cytokines, thus stimulating a complex network of cytokine signaling. Interleukin (IL)-1β, IL-8, monocyte chemoattractant protein (MCP-1), and granulocyte-colony stimulating factor (G-CSF) may aid in recruitment and activation of neutrophils at the maternal-fetal interface, which then secrete inflammatory cytokines and matrix metalloproteinases (MMPs). IL-1β, IL-6, and tumor necrosis factor (TNF)α can induce further production of MMPs by the chorioamniotic membranes and cervical smooth muscle cells. Simultaneously, IL-6, IL-2, IL-1α, IL-1β, and TNFα lead to the upregulation of prostaglandins by the amnion and decidua, either directly or indirectly through induction of cortisol. IL-1β and TNFα aid in maintaining prostaglandins through inhibition of prostaglandin dehydrogenases. This increase in prostaglandins results in myometrial contractions and the increase in MMPs weakens the chorioamniotic membranes, leading to premature rupture of membranes (PROM) and spontaneous preterm labor. Figure made using BioRender.

Chorioamnionitis is a heterogenous condition, with wide variation in the timing of exposure and an often unclear underlying cause of inflammation. Chorioamnionitis can have an infectious etiology^18–22^ or be caused by sterile inflammation.^18^ Pathogen-associated and/or damage-associated molecular patterns (PAMPs and DAMPs) are recognized by pathogen recognition receptors (PRRs), including toll-like receptors (TLRs), on both maternal-derived^23^ and fetal-derived^24,25^ placental cells. This initiates a consistent inflammatory cascade, with elevated levels of the pro-inflammatory cytokines and chemokines interleukin (IL)-1α,^26–28^ IL-1β,^29–33^ IL-2,^34^ IL-6,^29,33,35–37^ IL-8,^29,31,32,35,36^ tumor necrosis factor (TNF)α,^29,32,33,38^ monocyte chemoattractant protein (MCP-1)^36,39^, and granulocyte-colony stimulating factor (G-CSF)^30^ in the amniotic fluid and umbilical cord blood. This inflammatory response is believed to drive parturition and subsequent neonatal morbidities through a complex network of cytokine signaling (Fig. 2).^40–43^ Chorioamnionitis with fetal viability typically occurs during the developmental window from the late second to the end of the third trimester and this inflammatory exposure has long-term effects, including an increased risk of cerebral palsy, neurodevelopmental delay, asthma, and wheezing.^11,44,45^ Moreover, many of the neonatal morbidities associated with chorioamnionitis are both more common and more severe in males, including premature birth, late-onset sepsis, intraventricular hemorrhage, bronchopulmonary dysplasia, necrotizing enterocolitis, and neurobehavioral abnormalities.^46–50^ However, very few studies have investigated the sex-specific effects of chorioamnionitis on offspring outcomes.

As early as the first trimester, there are significant differences in sex hormone production between the two sexes. The male fetus begins to produce testosterone around 8–10 weeks post-fertilization, and the quantity of fetal testosterone in the amniotic fluid is 2- to 5-fold greater than that of a female fetus by 16 weeks post-fertilization.^51–54^ Placental architecture and function also vary considerably by sex.^55,56^ Many of the transcriptomic differences between male and female placentas include immune signaling genes,^57–64^ which could substantially alter the placental and fetal response to chorioamnionitis. Despite this, only a limited number of chorioamnionitis studies have investigated whether neonatal outcomes differ as a result of the sexually dimorphic nature of the human placenta and fetus. This review aims to synthesize current evidence on sex-specific outcomes following chorioamnionitis exposure in both humans and animal models, and to emphasize the importance of incorporating sex as a biological variable in perinatal research.

**Fig. 3:**
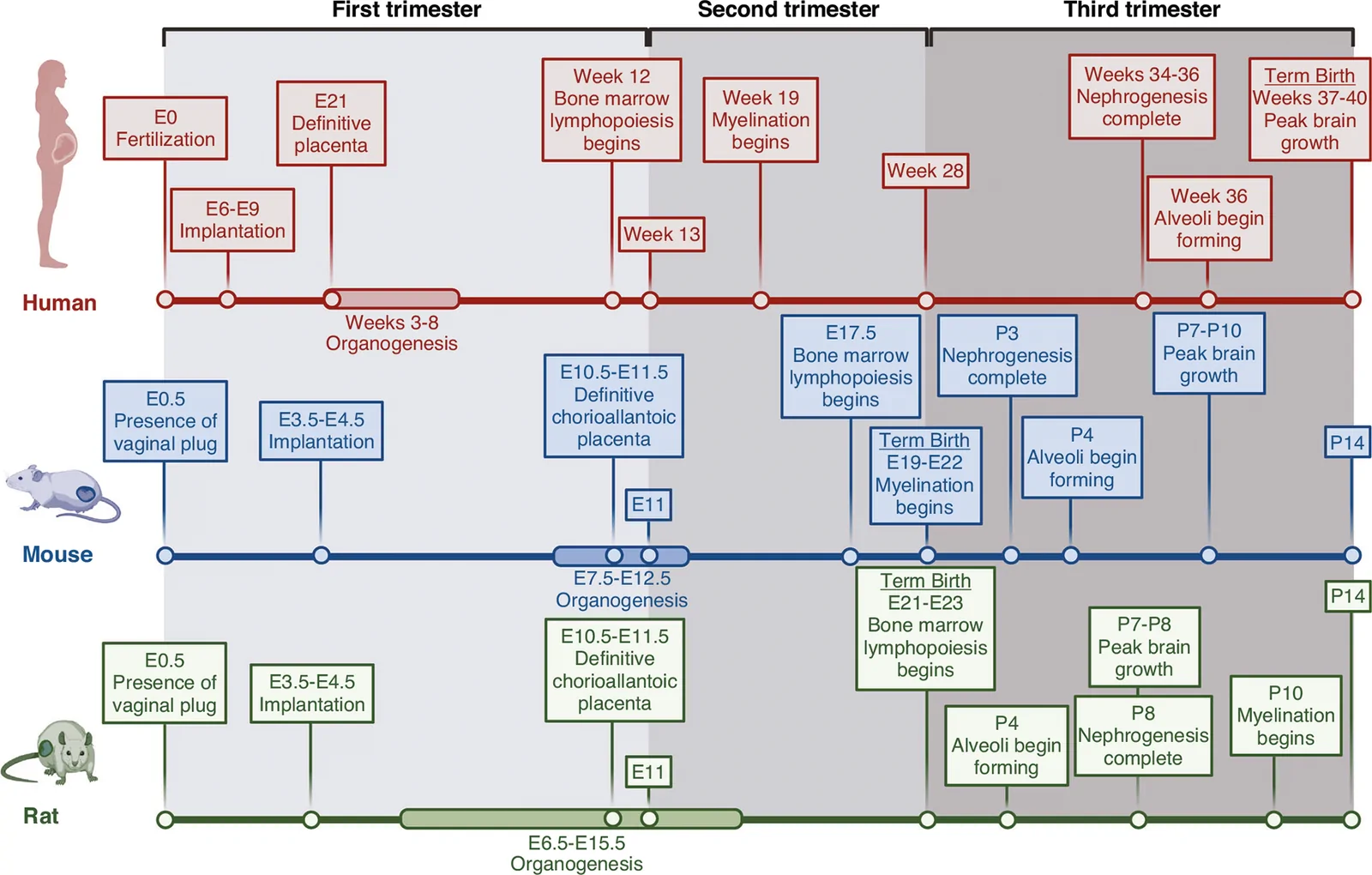
Trimester equivalents in mice and rats. The developmental timeline of gestation is similar between mice and rats but differs considerably from that of humans. Human gestation lasts for approximately forty weeks, while gestation in mice and rats lasts approximately twenty-one days. Many developmental milestones that begin early in human pregnancy do not begin until just before birth or even after birth in mice and rats, including bone marrow lymphopoiesis and myelination. Additionally, a substantial amount of lung and kidney development occurs prior to birth in humans, while these organs are still undergoing considerable developmental changes after birth in murine offspring. This delay makes the murine developmental equivalent of the human third trimester more closely in line with the first few postnatal weeks. Figure made using BioRender.

Few studies have investigated the role of sex in chorioamnionitis-related outcomes, and these studies have only emerged within the past two decades. Albeit difficult, the most clinically relevant method of studying chorioamnionitis is in humans. To date, there have been twelve studies investigating sex-specific differences in human placental inflammation, a full summary of which can be found in Table 1. In addition to these human studies, this review also describes twenty-four murine studies and one ovine study that investigated sex-specific differences in offspring outcomes following exposure to acute maternal immune activation (MIA). Murine models are among the most widely used systems to study intrauterine inflammation due to their low cost, hemochorial placentation, short gestational period, and amenability to genetic manipulation. Although these models enable rapid and detailed investigation of the mechanisms underlying perinatal inflammation-induced morbidities, their translational relevance is limited by species-specific gestational differences (Fig. 3)^65–72^ and fundamental differences in placental structure. While both humans and rodents possess a hemochorial placenta, in which fetal tissue is in direct contact with maternal blood, their placental architectures differ substantially.^73^ Human placentas are villous in structure, whereas rodent placentas are labyrinthine, with a defined junctional zone and an inverted visceral yolk sac that lacks a human counterpart.^73^ These structural differences limit the direct translatability of murine placental studies, although such models remain valuable for mechanistic insights into perinatal inflammation. As timing of gestational exposure is relevant to offspring outcomes, the murine findings have been organized both by outcome type and trimester equivalent. The full summaries of the murine studies can be found in Tables 2–8.

## RATES OF PLACENTAL INFLAMMATION

Two large human studies suggest that premature male neonates are equally as likely as females to have been exposed to acute inflammation or clinical chorioamnionitis (*n* = 437 [232 male, 205 female] and *n* = 446 [220 male, 226 female], respectively).^74,75^ This is in contrast to a study of successfully resuscitated neonates born at 23 weeks’ gestation by Miltaha et al. (*n* = 87), which observed a significantly higher rate of clinical chorioamnionitis among successfully resuscitated female compared to male neonates,^76^ but did not report rates among those who were not successfully resuscitated. Together, these studies suggest that the rate of acute placental inflammation likely does not differ between premature male and female neonates, although fetal age and sex-specific alterations in the maternal and fetal inflammatory responses may explain the differences in clinical presentation observed in the study by Miltaha et al (Table 1).^76^

Despite similar rates of acute inflammation, these two larger studies demonstrate that premature male neonates are more likely to have been exposed to chronic placental inflammation than females.^74,75^ Though males tended to have fewer chronic inflammatory lesions within the placenta,^74^ these lesions were more common,^74,76^ histologically more severe,^74^ and more likely to be localized at the implantation site.^74,76^ This was not consistently associated with an increase in chronic villitis, a finding that would be suggestive of maternal host-versus-graft disease (Table 1).^74,76^ This suggests an innate tendency for normal maternal tolerance toward the fetus to be reduced in the case of males, which may be driven by early immunogenic differences in male fetuses related to androgen exposure and the absence of a second X chromosome, potentially limiting inflammatory buffering within the placenta.

## MATERNAL AND FETAL INFLAMMATORY RESPONSES TO PLACENTAL INFLAMMATION

Although rates of acute placental inflammation did not differ by fetal sex, Goldenberg et al. reported that premature males were more likely to have placental bacterial cultures that were positive for *Ureaplasma urealyticum*^77^ and *Mycoplasma hominis*,^75^ both of which are known to cause chorioamnionitis (Table 1). This suggests that male placentas and/or fetuses may have a less robust antibacterial response during the third trimester. This aligns with the findings of Zein et al., which demonstrate a more robust inflammatory response in the arterial cord blood of premature females at the time of birth following acute placental inflammation, characterized by increased levels of fibroblast growth factor basic (FGF-basic), G-CSF, Il-1β, Il-1ra, Il-8, MIP-1α, and TNFα (Table 1).^78^ In this same study cohort, however, premature males exhibited what may represent a delayed or more sustained inflammatory response, with significantly higher peripheral blood TNFα levels in binned samples collected between twenty-four to seventy-two hours after birth compared with females.^78^ These sex-specific differences in cord blood cytokine concentrations do not align with the cytokine profiles measured in umbilical cord tissue lysates by Chehroudi et al., which contained variable amounts of cord blood and showed elevated cytokine concentrations in chorioamnionitis-exposed tissue without any sex-related differences (Table 1).^79^ Because cytokine levels are generally higher in blood than umbilical cord tissue following fetal exposure to chorioamnionitis, it is possible that the processing approach used in this study diminished or obscured any underlying sex-specific differences.^79^

To date, seven murine studies have investigated sex-specific differences in the initial inflammatory response to MIA (Table 2). These studies are either based on injection of lipopolysaccharide (LPS) or live *Streptococcus agalactiae* (group B streptococcus, GBS) into mouse or rat dams during the second trimester equivalent. Notably, male placentas demonstrate a more profound inflammatory response to MIA, characterized by earlier infiltration of macrophages (i.e., by three hours post-injection)^80^ and greater infiltration of polymorphonuclear cells at 72 h post-injection.^81,82^ This heightened cellular infiltration is accompanied by increased placental expression of pro-inflammatory cytokines at both early (i.e., 2 h post-injection) and late (i.e., 72 h post-injection) timepoints,^82,83^ as well as more extensive placental tissue necrosis (24 h post-injection).^83^ This increase in cellular infiltration and inflammation of the placenta may represent an increased maternal inflammatory response with male fetuses during chorioamnionitis, in contrast to the human study^84^ which suggests an increased fetal inflammatory response (i.e., via arterial cord blood) in females. This early acute response to MIA in male placentas is accompanied by increased systemic inflammation in male offspring at the later timepoint of 72 h post-injection,^82^ similar to what is observed in the human study, as well as a more robust neuroinflammatory response within the fetal brain.^83^ This includes earlier microglial activation (i.e., by 3 h post-injection),^80^ elevated cytokine mRNA expression at both early (i.e., 4 h post-injection) and late (i.e., 2+ days post-injection) timepoints,^85,86^ and loss of actively dividing (pHH3 +) radial glial cells at 2 h post-injection, indicating increased cortical hypoxia.^83^ However, female offspring also mount an early neuroinflammatory response to MIA, one that differs from males and involves the upregulation of distinct cytokines and metabolites within the fetal brain.^83,87,88^ Overall, these studies suggest that MIA may elicit a stronger initial maternal inflammatory response with male offspring, and a stronger initial fetal inflammatory response in female offspring. These sex-specific patterns highlight the need to investigate outcomes following MIA in males and females separately.

Two human studies by Konwar et al. investigated sex-specific epigenetic changes in the placenta and fetal membranes following chorioamnionitis exposure (Table 1).^89,90^ Epigenetics refers to heritable changes in gene expression without changes in the underlying gene sequence, and includes DNA methylation or acetylation, histone tail modifications, and microRNA expression (miRNAs). In their first study, Konwar et al. investigated genome-wide DNA methylation of CpG sites in 122 chorionic villus samples, sixteen chorion samples, and sixteen amnion samples.^89^ While they were unable to identify chorioamnionitis-associated methylation changes within the fetal membranes (potentially due to low sample number), they showed that chorioamnionitis was associated with sixty-six differentially methylated CpG sites in the chorionic villi, many of which were immune related.^89^ While there were no sex-specific differences in DNA methylation in the chorionic villi, the authors acknowledge that the study was likely not adequately powered to identify any differences that may exist.^89^ A follow-up study by Konwar et al. investigated the effect of chorioamnionitis on the placental expression of six miRNAs.^90^ They found that chorioamnionitis-exposed female placentas had decreased expression of miRNA-518b, which was not observed in male placentas.^90^ Certain pregnancy complications are associated with reduced placental miRNA-518b expression, including premature rupture of membranes (PROM) and intrauterine growth restriction.^91,92^ miRNA-518b expression is also associated with a polymorphism in *IL6* within this study and may play a role in neutrophil recruitment.^90^ Thus, this study suggests that there are placental epigenetic changes associated with chorioamnionitis that are fetal sex-specific and may affect the initial inflammatory response to chorioamnionitis.

## NEURODEVELOPMENTAL OUTCOMES

Much of the existing research on chorioamnionitis examines its impact on offspring neurodevelopment, and it is well established that exposure to chorioamnionitis increases the risk of cerebral palsy, intraventricular hemorrhage, and neurodevelopmental delay.^9,10^ Importantly, these conditions are both more prevalent and more severe in males,^46,49,50^ but few studies have investigated sex-specific differences in long-term neurodevelopmental outcomes following chorioamnionitis. We identified three human studies that investigated sex-specific differences in cerebral blood flow and/or brain metabolites following chorioamnionitis exposure and related these factors to neurodevelopmental scores at twelve months of age (using the Bayley III Scale of Infant and Toddler Development, Table 1).^93–95^ Additionally, we identified fifteen murine studies examining sex-specific differences in behavioral abnormalities in offspring following MIA (Table 3).

In a prospective cohort study of fifty-two term neonates, Koch et al. observed that chorioamnionitis-exposed males had significantly increased blood flow in the middle cerebral artery (MCA) compared to unexposed males, but that chorioamnionitis-exposed females had decreased blood flow in the MCA and anterior cerebral artery (ACA) compared to unexposed females.^93^ As cerebral blood flow generally increases at sites of inflammation, this may reflect a greater cerebral inflammatory response to chorioamnionitis in males, particularly within sites supplied by the MCA, which includes the motor cortex.^96^ Because inflammation of the MCA is linked to the development of cerebral palsy,^97^ this altered cerebral blood flow may put males at higher risk of developing cerebral palsy following chorioamnionitis. In this study, Bayley III composite scores at twelve months were not significantly altered by sex or histological chorioamnionitis.^93^ It is important to note, however, that Bayley III scores in infancy have limited sensitivity for predicting later motor function in childhood.^98,99^ Sex-specific patterns did emerge when cerebral blood flow was compared to the Bayley III sub-scores at twelve months of age. MCA blood flow was negatively correlated with problem-solving skills in males, whereas it was positively correlated with fine motor skill in females.^93^ Taken together, these findings indicate that exposure to histologic chorioamnionitis leads to sex-specific alterations in cerebral blood flow, which may have implications for long-term neurodevelopment.

A subsequent study examining a prospective cohort of twenty-four neonates born to mothers with clinical chorioamnionitis suggests that the combined effects of sex and chorioamnionitis on cerebral blood flow may vary according to gestational age.^94^ This study further supported the finding of Koch et al. that term chorioamnionitis-exposed males display decreased MCA resistance compared to females, but also found that preterm clinical chorioamnionitis-exposed males display higher basilar artery (BA) and MCA resistance compared to females.^93,94^ This indicates that chorioamnionitis results in sex-specific changes in cerebral blood flow that vary by gestational age at the time of exposure and may contribute to differential neurodevelopmental outcomes.

An additional study investigated brain metabolites in a prospective cohort of forty-four term neonates born to mothers with clinical chorioamnionitis accompanied by funisitis, defined as inflammation of the blood vessels within the umbilical cord.^95^ Although male and female infants demonstrated comparable stages and grades of funisitis, only males showed significant alterations in brain metabolites that correlated with the severity of umbilical cord inflammation.^95^ Males with more severe funisitis had significantly lower ratios of *N*-acetyl-aspartate/*N*-acetylaspartylglutamate (NAA) to choline (NAA/Cho) in the basal ganglia, with significantly higher ratios of myoinositol (mI) to NAA (mI/NAA) in the white matter.^95^ This pattern contrasts with normal *in utero* and postnatal development, which is characterized by an increasing NAA/Cho ratio and a decreasing mI/NAA ratio.^100–103^ Across both sexes, a lower NAA/Cho ratio was correlated with lower Bayley III composite motor scores at one year of age, and a higher mI/NAA ratio was associated with lower fine motor skills.^95^ This study also identified sex-specific associations between brain lactate ratios and Bayley III scores at one year of age.^95^ In females, lactate ratios within the white matter were consistently negatively associated with fine motor performance, whereas in males, lactate ratios in the basal ganglia showed consistent negative correlations with gross motor scores and motor composite scores.^95^ These findings suggest that males experience greater disruptions in typical developmental brain metabolite profiles following exposure to chorioamnionitis, which may place them at increased risk for movement disorders such as cerebral palsy. In support of this, children with cerebral palsy exhibit decreased NAA/Cho ratios and increased mI/NAA ratios within the basal ganglia.^104^

Taken together, these three human studies suggest that males experience enhanced neuroinflammation following chorioamnionitis, which is accompanied by alterations in cerebral blood flow and brain metabolites, particularly in regions critical for motor function. This may place male neonates at higher risk of developing cerebral palsy and neurodevelopmental impairment following chorioamnionitis, with potential life-long consequences. In contrast, alterations in cerebral blood flow and lactate ratios in females exposed to chorioamnionitis correlate with fine motor impairment, suggesting distinct motor impairments in females.

To our knowledge, there are no human studies that have investigated sex differences in long-term neurodevelopmental outcomes following chorioamnionitis. However, many studies of MIA in mice and rats have investigated behavioral abnormalities in adult offspring. These studies show that MIA-exposed offspring display behaviors characteristic of autism spectrum disorder (ASD), including decreased sociability,^105–108^ increased anxiety-like behaviors,^109,110^ and increased repetitive behaviors.^105,107,110^ Most of the investigations that subanalyzed these behavioral abnormalities by sex used models in which MIA was induced during the rodent equivalent of the second trimester, a developmental period in which corticogenesis is occurring.^111,112^ Most of these studies used a bacterial-based model of MIA (i.e., LPS or GBS administration to the dam), though two studies included a Poly(I:C)-based model (i.e., a viral mimetic). In general, these reports describe more pronounced behavioral abnormalities in male MIA-exposed offspring, with a notable, consistent decrease in sociability across different models.^81,83,84,113–118^ Additionally, in many of these studies, MIA-exposed males displayed increased anxiety-like behavior,^83,115,116,118–121^ with inconsistent findings regarding locomotor function and spatial memory. In contrast, MIA-exposed females rarely exhibited alterations in sociability, and studies reporting differences in females were less consistent. Together, these findings in humans and murine models provide strong evidence that chorioamnionitis induces enduring behavioral abnormalities, with moderate evidence that these abnormalities are more severe in males.

## ALTERATIONS IN CEREBRAL STRUCTURE

We identified eight murine studies that investigated sex-specific long-term alterations in cerebral structure following perinatal exposure to MIA (Table 4). These studies were primarily based on bacterial models of chorioamnionitis (i.e., administration of either LPS or live GBS to the dam) and were split evenly between mouse and rat models. These studies showed that perinatal inflammation caused alterations in cerebral structure in both male and female offspring, though the nature of these alterations varied by sex. Prior to weaning, both male and female MIA-exposed offspring had increased neuronal density in the hippocampus.^122^ However, only LPS-exposed males had increased spine density in hippocampal granule cells,^84^ a difference that may reflect impaired synaptic pruning. In adulthood, male MIA-exposed offspring had greater neuronal cell loss compared to females. This was reflected in reduced hippocampal neuron counts,^85^ decreased cellular density in the posterior cortex,^83^ reduced hippocampal volume,^123^ patches of axonal loss in the external capsule,^118^ thinning of the corpus callosum,^81^ thinning of the external capsule,^81^ thinning of the primary motor cortex,^121^ and enlarged lateral ventricles.^81,118^ However, both male and female MIA-exposed offspring exhibited cortical layer disorganization into adulthood. Notably, males had differences in excitatory and inhibitory neuronal density that were localized to specific cortical layers, whereas females displayed alterations that extended throughout broader regions of the cortex.^83^ In addition, both sexes demonstrated persistent cerebral damage into adulthood. This was evidenced by significant reductions in forebrain myelin basic protein,^85,121^ though one study reported this myelin loss only in females.^118^ Together, these findings indicate that while both sexes experience lasting structural brain changes following exposure to perinatal inflammation, the specific manifestations of this injury vary by sex.

## ALTERATIONS IN NEUROIMMUNITY

We identified eight murine studies that investigated sex-specific differences in long-term neuroinflammation and neuroimmunity in MIA-exposed offspring (Table 5). Each of these studies was based on administration of either LPS or live GBS to pregnant dams. Several studies indicate that MIA exposure alters baseline immune cell profiles in the adult brain of both sexes, including reduced mature oligodendrocyte numbers in the forebrain,^118^ increased whole-brain microglial density,^85^ and enhanced hippocampal gliosis.^114^ These cellular changes are accompanied by heightened inflammatory signaling, including elevated levels of TNFα and superoxide dismutase (SOD),^114^ as well as increased TNFα+ microglia throughout the brain.^85^

Evidence indicates that sex-specific neuroimmune alterations emerge early after MIA and persist throughout life. Prior to weaning, MIA-exposed males demonstrate a significant reduction in hippocampal *Cx3cr1* expression, a change not noted in females. In adulthood, MIA-exposed males demonstrate reduced expression of microglial markers,^124^ increased macrophage infiltration into the cortex,^123^ and a notable loss of Iba1+ microglia/macrophages in the corpus callosum.^121^ In contrast, MIA-exposed females show increased cortical *Slc1a2* expression,^124^ a potentially protective response,^125^ and reduced Iba1+ cell density in the external capsule.^118^ An adult immune challenge further highlights these sex-specific differences. MIA-exposed males display strong inflammatory gene induction across multiple brain regions, while females do not exhibit this heightened response.^85^ Collectively, these findings support persistent sex-specific neuroimmune phenotypes after MIA, with greater neuroinflammatory responses in adult males.

## ALTERATIONS IN BRAIN GENE, PROTEIN, AND METABOLITE EXPRESSION

Seven murine studies investigated how MIA altered brain expression of various genes, proteins, and metabolites in a sex-specific manner (Table 6). Three of these studies focused on the *Gad1* transcript or its encoded enzyme, glutamic acid decarboxylase 67 (Gad67). Gad67 is expressed in the murine brain during mid-to-late gestation and is the primary enzyme responsible for synthesizing γ-aminobutyric acid (GABA), a major inhibitory neurotransmitter.^126^ Altered GAD67 expression and localization have been documented in post-mortem brain tissue from individuals with schizophrenia and autism,^127^ both of which are associated with perinatal exposure to maternal inflammation.^128,129^ Of the three studies assessing Gad1/Gad67, two reported expression changes only in LPS-exposed males,^85,122^ while the other one found altered Gad67 levels in both LPS-exposed males and females.^114^ Notably, one study also detected increased density of Gad67+ cells in the ventral striatum oriens of Poly(I:C)-exposed females, but not males.^122^ This suggests that males and females may respond differently to MIA depending on whether the immune challenge is viral or bacterial.

Additional studies have reported sex-specific alterations in the expression of various neurotransmitters and their receptors in the adult brain following MIA exposure. Reduced whole-brain levels of 5-hydroxyindoleacetic acid (5-HIAA; the main metabolite of serotonin^130^) and reduced hippocampal levels of the N-methyl-D-aspartate (NMDA) receptor subunit NR2B^113^ were reported in both males and females exposed to LPS.^114^ In contrast, several studies observed neurotransmitter-related changes only in LPS-exposed males. These male-specific alterations included decreased hippocampal expression of glutamate,^113^ elevated hippocampal corticosterone levels,^113^ reduced hippocampal expression of the glucocorticoid receptor,^113^ and increased amygdala expression of *Drd2* (dopamine receptor 2)^85^ compared with control males. In a separate MIA model using Poly(I:C), males also showed increased ventral hippocampal expression of *Oxtr* (oxytocin receptor), *Crh* (corticotropin releasing hormone), *Crhr1* (corticotropin releasing hormone receptor 1) and *Nr3c1* (nuclear receptor subfamily 3 group C member 1) relative to control males.^117^ By comparison, Poly(I:C)-exposed females only displayed a reduction in ventral hippocampal *Oxtr*.^117^ This further supports the idea that viral and bacterial forms of MIA produce distinct sex-specific effects in offspring.

Finally, one study examined histone tail modifications in the hippocampus, specifically focusing on H3K9me3 and H4K12ac,^131^ as both marks have previously been shown to change after perinatal LPS exposure.^132,133^ The researchers reported that at 3 months of age, only LPS-exposed males showed significantly increased hippocampal levels of H3K9me3 and significantly decreased levels of H4K12ac.^131^ By 13 months of age, however, both LPS-exposed males and females exhibited altered levels of these histone tail modifications compared with their respective controls but males demonstrated higher levels of H3K9me3 compared to females.^131^ These findings suggest a more pronounced phenotype in LPS-exposed males that may result in earlier cognitive decline.

## PULMONARY OUTCOMES

It is well-established that chorioamnionitis increases the risk of bronchopulmonary dysplasia, childhood asthma, and childhood wheezing,^14,44^ despite a reduction in risk of respiratory distress syndrome.^134^ In the general population, bronchopulmonary dysplasia, childhood asthma, and childhood wheezing are more prevalent in males.^44,48,135,136^ However, in adulthood, asthma and wheezing are more prevalent in females.^135,136^ This suggests a direct role for hormonal differences between males and females in the development of chronic lung disease. However, to our knowledge, only one human study and one ovine study to date have investigated sex differences in pulmonary parameters following chorioamnionitis exposure. An ovine study comparing lung gas volumes in preterm lambs exposed to intra-amniotic injection of LPS found that LPS-exposed females had significantly increased lung gas volumes compared to LPS-exposed males.^137^ This suggests that chorioamnionitis induces lung maturation more effectively in LPS-exposed females compared to LPS-exposed males. In this model, there were not baseline differences in lung compliance between control preterm males and females, suggesting that chorioamnionitis exposure drives the sex differences.^137^ Previous ovine models have demonstrated that fetal LPS exposure quickly increases surfactant expression within the lung,^138^ which likely drives the increase in lung compliance within this study. In the fetal lung, androgens inhibit surfactant production,^139,140^ which may act to negate the pulmonary effects of LPS exposure in males. Additionally, in a prospective cohort of ninety-five preterm human neonates, Jones et al. observed that females exposed to histologic chorioamnionitis had significantly reduced lung function during the first postnatal year compared to unexposed females, an effect that was not observed in males (Table 1).^141^ Importantly, unexposed preterm females had significantly better lung function than unexposed preterm males, with chorioamnionitis exposure appearing to narrow this physiologic gap between the sexes.^141^ This suggests that preterm females may be particularly vulnerable to long-term lung dysfunction following chorioamnionitis exposure. However, because male lung development lags behind that of females,^142^ the impact of exposure may vary depending on the gestational age at which it occurs. Together, these two studies suggest that chorioamnionitis induces accelerated fetal maturation of the lungs within females, but differential alterations in immune programming may account for the female-specific reduction in lung function at one year of age.

## CARDIAC OUTCOMES

Chorioamnionitis increases the risk of patent ductus arteriosus and fetal cardiac concentric hypertrophy,^143,144^ both of which are associated with cardiovascular dysfunction. Additionally, young adults born prematurely have increased left and right ventricular mass, which is associated with both systolic and diastolic dysfunction.^145,146^ As prematurity is highly associated with chorioamnionitis, these findings suggest that chorioamnionitis remodels cardiac architecture and physiology during fetal development, with life-long implications for cardiac function. Moreover, female young adults born preterm had a greater ratio of right ventricular mass to end diastolic volume compared to males, indicating a greater degree of right ventricular hypertrophy.^145^ Despite these findings, very little research has investigated sex differences in cardiac function following chorioamnionitis. In the previously noted human study of twenty-four infants born to mothers with clinical chorioamnionitis, Jenkins et al. reported a significantly lower ejection fraction in exposed male neonates compared to exposed females (Table 1).^94^ Unfortunately, this study did not include unexposed controls, making it difficult to draw firm conclusions. It remains possible that baseline differences in ejection fraction exist between male and female neonates independent of chorioamnionitis exposure, as has been reported in adults.^147^ Additionally, in a murine study, Ushida et al. reported that MIA-exposed adult female offspring had a significant elevation in *Mef2c* expression in the left ventricle.^148^ This suggests a predisposition for cardiac complications in MIA-exposed females, as the cardiac developmental gene *Mef2c* has been associated with cardiac hypertrophy,^149^ which would align with the human studies demonstrating increased right ventricular hypertrophy in female adults born prematurely.

## METABOLIC OUTCOMES

Increased severity of chorioamnionitis has been linked to increased fat mass accretion in preterm infants,^150^ but few studies have investigated how sex plays a role in metabolic dysfunction following chorioamnionitis. In a retrospective cohort study of 4220 preterm infants from the Neonatal Research Network of Japan, Ushida et al. found that chorioamnionitis was associated with accelerated postnatal weight gain at three years of age in female, but not male, offspring (Table 1).^151^ This suggests that females exposed to chorioamnionitis may face a higher risk of childhood obesity and, consequently, an elevated risk of developing metabolic syndrome in adulthood. The large sample size lends power to this finding and highlights the need for more research in this area.

Although numerous studies report reduced weight in MIA-exposed neonates, relatively few have examined long-term metabolic consequences. We identified four studies that investigated sex-based differences in long-term metabolic outcomes following MIA exposure (Table 7). Two of these studies indicate that MIA-exposed males have worse metabolic outcomes compared to females,^148,152^ though one study found that LPS-exposed females were more susceptible to metabolic disease when challenged with a western-style diet.^124^ Collectively, these studies identified glucose intolerance,^148,152^ insulin resistance,^124,152^ increased adiposity,^86,124,148,152^ reduced muscle mass,^86^ and elevated circulating leptin levels^86,152^ in adult male offspring exposed to MIA. In contrast, most studies found that comparable metabolic alterations only emerged in MIA-exposed adult female offspring when they were placed on a high-fat, western-style diet.^124^ Overall, the evidence suggests that long-term metabolic consequences of MIA are more pronounced in males unless females encounter additional metabolic challenges, conflicting with the large-scale human study.

## MISCELLANEOUS SYSTEMIC OUTCOMES

We identified one murine study that examined sex-specific differences in additional systemic outcomes in offspring following MIA (Table 8). This study found that MIA-exposed female offspring had significant reductions in red blood count and hematocrit and increased clot formation time, which was not noted in males.^148^ The authors also investigated transgenerational outcomes by breeding 10–14-week-old MIA-exposed females with non-experimental males. At embryonic day 17.5, they noted decreased Glut1 (glucose transporter-1) and increased CD68+ cells (macrophages) in the mesometrial triangle, junctional zone, and labyrinth.^148^ This suggests that adult female offspring exposed to MIA experience heightened uteroplacental inflammation during pregnancy with a decreased ability to transfer glucose to the fetus, which likely contributes to the increased incidence of fetal growth restriction observed in this group.

## LIMITATIONS

The findings of the studies detailed in this review have some notable limitations. Most of the human studies include patients from a single institution, which may limit the generalizability of the findings. Additionally, the gestational age of the included patients differs widely by study and the definitions of clinical and histological chorioamnionitis vary by institution, highlighting the need for standardized definitions. Many of the human studies also include a relatively small sample size, which may not be fully powered to identify subtle sex-specific differences. While the murine studies use well-established models of chorioamnionitis, each model has its own limitations. Intraperitoneal, intrauterine, subcutaneous, and tail vein injections of dams with an immunogen do not model the natural route of chorioamnionitis, which is thought to most often occur by an ascending infection. Intrauterine injections are performed via mini laparotomy, which introduces the variables of surgery and anesthesia. Additionally, all the models result in some level of systemic inflammation in the dam, which may only mimic more severe cases of histological chorioamnionitis. Finally, these models employ a range of immunogens that each represent only bacterial, viral, or sterile chorioamnionitis, thereby limiting the generalizability of the findings.

## CONCLUSIONS

Chorioamnionitis is a common pregnancy complication that is associated with an increased risk of numerous neonatal and childhood morbidities. Placental biology shows substantial sex-based differences beginning early in gestation, and both human and murine studies show that the initial inflammatory response to placental inflammation differs markedly by fetal sex. Despite this, few studies have evaluated the effect of fetal sex on outcomes following perinatal inflammatory exposure. In this review, we evaluated thirty-seven studies that directly assessed sex-specific offspring outcomes after exposure to perinatal inflammation. The studies detailed here suggest that there may be a more robust maternal inflammatory response within male placentas, but a more robust fetal inflammatory response within females. This is accompanied by residual systemic inflammation within males, but not females, by 72 h following exposure. This sustained inflammation may contribute to the heightened neuroinflammatory profile observed in males, which is associated with reductions in gross motor and composite neurodevelopmental scores in humans, as well as decreased sociability in murine offspring. In contrast, females demonstrate a distinct neuroinflammatory profile, with alterations that correlate with deficits in fine motor function in humans, but without consistent behavioral abnormalities in murine studies. Beyond neurodevelopmental outcomes, the available evidence suggests that females exposed to chorioamnionitis may be at increased risk for long-term pulmonary dysfunction and cardiac hypertrophy. Additionally, large-scale human data indicate that exposed females may have greater susceptibility to childhood obesity and subsequent metabolic dysfunction in adulthood, whereas murine studies suggest that metabolic syndrome may be more prevalent in exposed males. Collectively, these studies provide evidence in both humans and animal models that perinatal inflammation elicits sex-specific responses that lead to divergent short- and long-term outcomes. These studies underscore the importance of incorporating both fetal sex and chorioamnionitis exposure into perinatal research and highlight the need for further studies focused on sex-specific mechanisms and outcomes.

## Figures and Tables

**Table 1.** Sex-specific outcomes following human chorioamnionitis.

Study	Outcome Type	Study Type	Sample Size	Age	Method	Measurement	Findings	Comparison Group
Ghidini et al.^74^	Rates of placental inflammation	Case control study of neonates born between 22 and 32 weeks’ gestation at the University of Connecticut Medical Center	437 (232 male, 205 female)	Males: 28.1 ± 2.4 weeks’ gestation Females: 28.1 ± 2.4 weeks’ gestation	Histological evidence of acute or chronic placental inflammation following a standard protocol^2^ by a blinded pathologist	Number of acute placental inflammatory lesions	No difference between sexes	-
						Number of chronic placental inflammatory lesions	↑ Female offspring	Male offspring
						Severity of chronic placental inflammation (via lesion score)	↑ Male offspring	Female offspring
						Relative risk of chronic inflammation at the basal plate	↑ Male offspring	Female offspring
						Relative risk of villitis of anchoring villi	↑ Male offspring	Female offspring
						Chronic inflammation at all other placental locations	No difference between sexes	-
Goldenberg et al.^75^	Rates of placental inflammation; placental and cord blood culture	Secondary evaluation of a prospective study of neonates born between 23 and 32 weeks’ gestation (the Alabama Preterm Birth Study)	446 placentas (220 male, 226 female) 343 umbilical cord blood samples	Males: 28.5 ± 2.3 weeks’ gestation Females: 28.6 ± 2.2 weeks’ gestation	Rate of spontaneous delivery	Rate of spontaneous delivery	↑ Male offspring	Female offspring
					Rate of clinical chorioamnionitis (as diagnosed by the attending obstetrician)	Rate of clinical chorioamnionitis	No difference between sexes	-
					Histological evidence of acute or chronic inflammation following a standard protocol^4^ by a blinded pathologist	Odds ratio of chronic lymphoplasmacytic inflammation	↑ Male offspring	Female offspring
						Rate of acute placental inflammation	No difference between sexes	-
						Rate of chronic villitis	No difference between sexes	-
					Cord blood IL-6 levels (via ELISA)	IL-6 levels	No difference between sexes	-
					Placental and cord blood culture for *Ureaplasma urealyticum* or *Mycoplasma hominis*	Rate of positive placental culture	↑ Male offspring	Female offspring
						Rate of positive cord blood culture	No difference between sexes	-
Miltaha et al.^76^	Rates of placental inflammation	Retrospective cohort of neonates born between 23^0/7^-23^6/7^ weeks’ gestation at Loyola University Medical Center	87 (did not report the number of males and females)	23^0/7^–23^6/7^ weeks’ gestation Mean gestational age: 23^4/7^ weeks	Clinical chorioamnionitis defined as follows: maternal fever (≥ 100.4 °F for ≥ 1 hour), foul-smelling amniotic fluid, and sustained fetal tachycardia (> 160bpm for ≥ 1 hour)	Rate of clinical chorioamnionitis	↑ Female offspring	Male offspring
Zein et al.^78^	Maternal and fetal inflammatory responses to placental inflammation	Prospective cohort of neonates born 23^0/7^-28^6/7^ weeks’ gestation at the Foothills Medical Center in Calgary, Alberta	42 placentas 12 with acute inflammation (5 males, 7 females)	25 (26–27) weeks’ gestation Blood collected at birth, 24–72 h after birth, and 21–28 days after birth	Umbilical cord blood levels of 27 cytokines (via Bio-Rad Multiplex Immunoassay System)	FGF-basic	↓ Chorioamnionitis-exposed males	Chorioamnionitis-exposed females
						G-CSF	↓ Chorioamnionitis-exposed males	Chorioamnionitis-exposed females
						IL-1β	↓ Chorioamnionitis-exposed males	Chorioamnionitis-exposed females
						IL-1 Rα	↓ Chorioamnionitis-exposed males	Chorioamnionitis-exposed females
						IL-8	↓ Chorioamnionitis-exposed males	Chorioamnionitis-exposed females
						MIP-1α	↓ Chorioamnionitis-exposed males	Chorioamnionitis-exposed females
						TNFα	↓ Chorioamnionitis-exposed males	Chorioamnionitis-exposed females
					Peripheral blood levels of 27 cytokines between 24–72 h after birth (via Bio-Rad Multiplex Immunoassay System)	TNFα	↑ Chorioamnionitis-exposed males	Chorioamnionitis-exposed females
Chehroudi et al.^79^	Maternal and fetal inflammatory responses to placental inflammation	Human umbilical cords from the Hawaii Biorepository	280 umbilical cords (170 females, 210 males)	38.3 ± 2.3 (25–42) weeks’ gestation	Umbilical cord tissue lysate cytokine levels (via multiplex ELISA)	IL-1β	↑ Chorioamnionitis-exposed neonates -No difference between sexes	Unexposed neonates
						IL-6	↑ Chorioamnionitis-exposed neonates -No difference between sexes	Unexposed neonates
						TNFα	↑ Chorioamnionitis-exposed neonates No difference between sexes	Unexposed neonates
						IL-8	↑ Chorioamnionitis-exposed neonates -No difference between sexes	Unexposed neonates
						IL-10	↑ Chorioamnionitis-exposed neonates -No difference between sexes	Unexposed neonates
						VEGFA	↓ Chorioamnionitis-exposed neonates -No difference between sexes	Unexposed neonates
Konwar et al.^89^	Maternal and fetal inflammatory responses to placental inflammation	Discovery cohort & validation cohort at the Children’s and Women’s Health Center of British Columbia	Discovery cohort: 44 placentas with 16 matched amnion and chorion samples 22 with acute histologic chorioamnionitis (11 male, 11 female) 22 without histologic chorioamnionitis (13 male, 9 female) Validation cohort: 78 chorionic villi samples 42 with acute histologic chorioamnionitis (24 male, 18 female) 36 without histologic chorioamnionitis (21 male, 15 female)	Discovery cohort: Acute chorioamnionitis: 28–36 (mean 30.74) weeks’ gestation Without chorioamnionitis: 28–36.7 (mean 32.28) weeks’ gestation Validation cohort: Acute chorioamnionitis: 20–41 (mean 26.83) weeks’ gestation Without chorioamnionitis: 20–39 (mean 29.2) weeks’ gestation	DNA methylation at 866,895 CpGs of the chorionic villi (via the Illumina Infinium HumanMethylationEPIC BeadChip [850 K array])	Methylation of cg21962324 *(IRX2)*	↑ Chorioamnionitis-exposed neonates -No difference between sexes	Unexposed neonates
						Methylation of cg11340524 *(RAB27A)*	↓ Chorio-amnionitis-exposed neonates -No difference between sexes	Unexposed neonates
						Methylation of cg01276475 *(RIMS1)*	*↓* Chorioamnionitis-exposed neonates -No difference between sexes	Unexposed neonates
Konwar et al.^90^	Maternal and fetal inflammatory responses to placental inflammation	Human placentas from University of British Columbia Children’s and Women’s Center	57 placentas: 33 with acute histologic chorioamnionitis (16 male, 17 female) 24 without histologic chorioamnionitis (13 male, 11 female)	Acute chorioamnionitis: 18–36 weeks’ gestation Without chorioamnionitis: 22.6–40.7 weeks’ gestation	Placental miRNA expression (via RT-qPCR)	Placental miR-338–3p expression	↑ Chorioamnionitis-exposed males	Unexposed males
							↑ Chorioamnionitis-exposed females	Unexposed females
						Placental miR-518b expression	*↓* Chorioamnionitis-exposed females	Unexposed females
Koch et al.^93^	Neurodevelopmental outcomes	Prospective cohort of neonates born ≥37 weeks’ gestation with clinical and/or histological chorioamnionitis at the Medical University of South Carolina	52 mothers: 7 with clinical chorioamnionitis (3 male, 4 female) 28 with histologic chorioamnionitis (15 male, 13 female) 17 controls (7 male, 10 female)	≥37 weeks’ gestation Bayley III scoring performed at a mean age of 11.5 ± 0.7 months (11.0–13.9)	Measures of cerebral blood flow (via cranial Doppler ultrasound)	MCA TAMX	↑ Chorioamnionitis-exposed males	Unexposed males
							↑ Chorioamnionitis-exposed males	Chorioamnionitis-exposed females
							*↓* Chorioamnionitis-exposed females	Unexposed females
						MCA CRI	*↓* Chorioamnionitis-exposed males	Chorioamnionitis-exposed females
						ACA CRI	*↓* Chorioamnionitis-exposed males	Chorioamnionitis-exposed females
					Correlation of cerebral blood flow and resistance measures with Bayley III scores	MCA TAMX	(−) correlation with problem solving in males	-
							( + ) correlation with fine motor skills in females	-
						ACA TAMX	(−) correlation with composite cognitive score in males	-
							(−) correlation with problem solving in males	-
							(−) correlation with personal-social skills in males	-
						BA CRI	(−) correlation with personal-social skills in females	-
Jenkins et al.^94^	Neurodevelopmental outcomes; cardiac outcomes	Prospective cohort of mothers at or greater than 24 weeks’ gestation with clinical signs of chorioamnionitis at the Medical University of South Carolina Part of a randomized control trial for N-acetylcysteine following clinical chorioamnionitis	24 (13 male, 11 female)	≥24 weeks’ gestation Doppler blood flow & echocardiogram during the first 48 postnatal hours	CRI (via cranial Doppler ultrasound)	BA CRI	↑ Preterm chorioamnionitis-exposed males	Preterm chorioamnionitis-exposed females
							↓ Term chorioamnionitis-exposed males	Term chorioamnionitis-exposed females
						MCA CRI	↑ Preterm chorioamnionitis-exposed males	Preterm chorioamnionitis-exposed females
							↓ Term chorioamnionitis-exposed males	Term chorioamnionitis-exposed females
					EF (via echocardiogram read by a single pediatric cardiologist)	EF	*↓* Chorioamnionitis-exposed males	Chorioamnionitis-exposed females
Johnson et al.^95^	Neurodevelopmental outcomes	Prospective cohort of neonates ≥37 weeks’ gestation born to mothers with clinical chorioamnionitis at the Medical University of South Carolina and with histologically confirmed funisitis	43 (19 females, 24 males) born to mothers with clinical chorioamnionitis (defined as maternal fever ≥38°C in the presence of rupture of membranes or two of the following: uterine tenderness, maternal white blood cell count >15,000 cells/mm, fetal tachycardia >160 bpm, or malodorous amniotic fluid)	Males: 39.5 ± 1.3 weeks’ gestation Females: 39.5 ± 1.2 weeks’ gestation Magnetic resonance spectroscopy (MRS) scans 1.5 ±0.9 (0.2–4.0) weeks after birth, with a gestational age of 38–44 weeks Bayley III scoring at 11.9 months (11.0–13.2)	Histologic evidence of funisitis	Presence of funisitis	No difference between sexes	-
					Brain metabolite concentrations (via Magnetic Resonance Spectroscopy)	NAA/Cho ratio in the basal ganglia	↓ Males with severe funisitis	Males with mild funisitis
						mI/NAA ratio in the white matter	↑ Males with severe funisitis	Males with mild funisitis
					Correlation of brain metabolite ratios with Bayley III scores	Lac ratio in the white matter	(−) correlation with fine motor scores in females	-
						Lac ratio in the basal ganglia	(−) correlation with gross motor scores in males	-
						Lac/NAA ratio in the basal ganglia	(−) correlation with motor composite scores in males	-
Jones et al.^141^	Pulmonary outcomes	Prospective cohort of infants born less than 37 weeks’ gestation in Potto Alegre, Brazil	95 (43 male, 52 female)	Males: 34.0 (24–36.8) weeks’ gestation Females: 34.6 (26–36.8) weeks’ gestation Lung function tests measured in the first postnatal year, after 40 weeks post-conceptual age	Lung function test using the raise volume-rapid thoracic compression technique	FVC	No difference between sexes	-
						FEV_0.5_	*↓* Chorioamnionitis-exposed females	Unexposed females
						FEF_25–75_	↓ Chorioamnionitis-exposed males	Chorioamnionitis-exposed females
							*↓* Chorioamnionitis-exposed females	Unexposed females
						FEF_50_	*↓* Chorioamnionitis-exposed males	Chorioamnionitis-exposed females
							*↓* Chorioamnionitis-exposed females	Unexposed females
Ushida et al.^151^	Metabolic outcomes	Multicenter, retrospective cohort study of preterm infants born from 2003–2017 who weighed ≤ 1500 g and were born <32 weeks’ gestation in the Neonatal Research Network of Japan	4220 infants 2110 with histologic chorioamnionitis (1,135 males, 975 females) 2110 without chorioamnionitis (1,135 males, 975 females)	With chorioamnionitis: 27.3 ± 2.2 weeks’ gestation Without chorioamnionitis: 27.3 ± 2.2 weeks’ gestation	Z-scores of height and weight at birth and 3 years of age based on age- and sex-specific reference standards using software provided by the Japanese Society for Pediatric Endocrinology	Mean z-scores for weight	↑ Chorioamnionitis-exposed females	Unexposed females

*ACA* anterior cerebral artery, *BA* basilar artery, *Cho* choline, *CpGs* cytosine-phosphate-guanosine dinucleotides, *CRI* corrected resistive index, *EF* ejection fraction, *ELISA* enzyme-linked immunosorbent assay, *FEF*_25–75_ forced expiratory flow between 25% and 75% of forced vital capacity, *FEF*50 forced expiratory flow at 50%of forced vital capacity, *FEV*0.5 forced expiratory volume in 0.5 s, *FVC* forced vital capacity, *Lac* lactate, *MCA* middle cerebral artery, *mI* myoinositol, *miRNA* microRNA, *NAA* N-acetyl-aspartate/N-acetylaspartylglutamate, *RT-qPCR* reverse transcription quantitative polymerase chain reaction, *TAMX* time-averaged maximum velocity.

**Table 2.** Initial inflammatory response to murine maternal immune activation.

Trimester Equivalent	Study	Study model	Mouse or rat strain	Age at outcome	Method	Measurement	Finding	Comparison Group
**Early Second**	Braun et al.^83^	Intraperitoneal injection of dams with LPS (30 μg/kg or 60 μg/kg, *E.coli* [strain not specified], Sigma Aldrich) on E12.5 *All pups were fostered at birth to wild-type, naïve dams	C57BL/6 J (Jackson Laboratories)	E12.5 (2 h after injection)	Placental cytokine levels (via ELISA)	TNFα	↑ LPS-exposed males	Control males
						CXCL1	↑ LPS-exposed males	LPS-exposed females
					Fetal brain cytokine levels (via ELISA)	CXCL10	↑ LPS-exposed females	Control females
					Fetal brain hypoxia (via pimonidazole staining on IF; pimonidazole was IP injected 15 min before LPS)	Percent area of cortical hypoxia (as measured by ratio of pimonidazole/DAPI)	↑ LPS-exposed males	LPS-exposed females
					Density of radial glial cells within the cortex (via IF)	pHH3+ nuclei density in the cortex	↓ LPS-exposed males	Control males
				E13.5 (24 h after injection)	Placental pathology (via H&E)	Placental tissue necrosis	↑ LPS-exposed males	Control males
						Placental weight gain	↓ LPS-exposed males	Control males
**Late Second**	Makinson et al.^85^	Intrauterine injection of dams with LPS (50 μg, *E. coli* 055: B5, Sigma Aldrich) on E15 via mini laparotomy under isoflurane anesthesia	CD-1 outbred mice from Charles River Laboratories	E17.5	Gene expression in the frontal poles of the fetal brain (via qPCR)	*Il1b*	↑ LPS-exposed males	Control males
				P2	Whole brain gene expression (via qPCR)	*C1q*	↑ LPS-exposed males	Control males
						*C3*	↑ LPS-exposed males	Control males
							↓ LPS-exposed females	Control females
						*Tgfb1*	↑ LPS-exposed males	Control males
						*Mbp*	↓ LPS-exposed offspring	Control offspring
							↑ Male offspring	Female offspring
						*Cnp*	↓ LPS-exposed offspring	Control offspring
							↑ Male offspring	Female offspring
	Brown et al.^87^	Intrauterine injection of LPS (50 μg, *E. coli* 055: B5, Sigma Aldrich) on E15 via mini laparotomy under isoflurane anesthesia	CD-1 mice from Charles River Laboratories	P2	Metabolic profiling of the postnatal brain (via UPLC-MS/MS)	Fatty acids (fold change) Palmitoleate (1.30) Stearate (1.42) Arachidonate (1.62) Pimelate (1.45) Adrenoylcamitine (1.59) 2-hydroxipalmitate (1.55) 2-hydroxystearate (1.44) Glycerophosphoserine (1.25) 1,2-dioleoyl-GPC (1.29) 1-oleoyl-2-arachidonoyl-GPE (1.12)	↑ LPS-exposed males	LPS-exposed females
						Metabolites involved in metabolism of sphingomyelins, diacylglycerols, and plasmalogens (fold change) Glycerophosphoserine (1.19) 1-palmitoyl-2-arachidonoyl-GPE (1.10) 1-oleoyl-2-arachidonoyl-GPE (1.12) 1-(1-enyl-palmitoyl)-2-arachidonoyl-GPE(1.11) Palmitoyl-arachidonoyl-glycerol (1.27) Sphingomyelin (1.27) Sphingomyelin (1.36) •Sphingomyelin (1.31) 2-oleoylglycerol (0.44)	↑ LPS-exposed females	LPS-exposed males
	Allard et al.^81^	Intraperitoneal injection of dams with live serotype Ia *Streptococcus agalactiae* (GBS, 10^8^-10^9^ CFU/100 μL saline) on E19	Lewis rats from Charles River Laboratories	E22 (72 h post-injection)	Placental pathology (via H&E)	PMN infiltration within the labyrinth compartment	↑ GBS-exposed males	GBS-exposed females
	Allard et al.^82^	Intraperitoneal injection of dams with live serotype la *Streptococcus agalactiae* (G8S, 10^8^-10^9^ CFU/100 μL saline) on E19	Lewis rats from Charles River Laboratories	E22 (72 h post-injection)	Placental pathology (via H&E)	PMN infiltration within the labyrinth compartment	↑ GBS-exposed males	GBS-exposed females
					Placental levels of inflammatory cytokines (via ELISA)	CXCL1	↑ GBS-exposed males	GBS-exposed females
						S100A9	↑ GBS-exposed males	GBS-exposed females
						IL-1β	↑ GBS-exposed males	GBS-exposed females
					Fetal serum levels of inflammatory cytokines (via ELISA)	IL-1β	↑ GBS-exposed males	Control males
	Chin et al.^86^	Intraperitoneal injection of dams with LPS (20 μg/kg, *Salmonella typhimuriutn*, Sigma Aldrich) on E16	C57BL/6 J mice from the Jackson Laboratories	E16 (4 h after injection)	Inflammatory gene expression in the placentas, fetal membranes, adjacent myometrium, and fetal brains (via RT-qPCR)	*Il10* in fetal brain	↑ LPS-exposed males	LPS-exposed females
						*Il1a* in myometrium	↑ LPS-exposed males	LPS-exposed females
						*Il1b* in myometrium	↑ LPS-exposed males	LPS-exposed females
						*Il6* in myometrium	↑ LPS-exposed males	LPS-exposed females
						*Il10* in myometrium	↑ LPS-exposed males	LPS-exposed females
	Na et al.^80^	Intrauterine injection of dams with LPS (25 μg*/*100 μL PBS, *E. coli* O55:B5, Sigma Aldrich) on E17 via mini laparotomy under isoflurane anesthesia	CD-1 mice from Charles River Laboratories	3- or 6-h post injection	Quantification of placental macrophages with frequency of subtypes (via flow cytometry)	Total placental macrophages at 3 h post-injection	↑ LPS-exposed males	Control males
						HBCs at 6 h post-injection	↑ LPS-exposed males	LPS-exposed females
						MIMs at both 3- and 6-hours post-injection	↑ LPS-exposed males	Control males
						M2 HBCs at 3-h post-injection	↓ LPS-exposed males	Control males
						M2 HBCs at 6-h post-injection	↓ LPS-exposed males	LPS-exposed females
						M2 MIMs at both 3- and 6-h post-injection	↑ LPS-exposed females	Control females
					Frequency of brain microglia subtypes (via flow cytometry)	M1 microglia at 3-hours post-injection	↑ LPS-exposed males	Control males
						M2 microglia at 3-hours post-injection	↓ LPS-exposed males	Control males

*DAPI* 4′,6-diamidino-2-phenylindole, *ELISA* enzyme-linked immunosorbent assay, *GBS* group B streptococcus, *H&E* hematoxylin and eosin staining, *HBCs* Hofbauer cells, *IF* immunofluorescence, *LPS* lipopolysaccharide, *MIMs* maternal inter-labyrinth macrophages, *PBS* phosphate buffered saline, *PMN* polymorphonuclear cell, *qPCR* quantitative polymerase chain reaction, *RT-qPCR* reverse transcription quantitative polymerase chain reaction, *UPLC-MS/MS* ultra-performance liquid chromatography-tandem mass spectrometry.

**Table 3.** Behavioral outcomes following murine maternal immune activation.

Trimester equivalent	Study	Study model	Mouse or rat strain	Age at outcome	Method	Measurement	Findings	Comparison Group
**First**	Connors et al.^113^	Intraperitoneal injection of dams with LPS (100 μg/kg, *E. coli* 026:B6) on E11	Sprague-Dawley rats from Charles River Laboratories	P40	Social interaction test	Time spent in social engagement (sociability)	↓ LPS-exposed males	Control males
					Object-in-place test	Ability to discriminate (spatial memory)	↓ LPS-exposed males	Control males
	Solmaz et al.^114^	Intra peritoneal injection of dams with LPS (100 μg/kg, *E. coli* O127:B8) on E10	Sprague-Dawley rats from the Experimental Animal Laboratory of Bilim University	P50	3 Chamber Sociability Test	Time spent with strange mouse (sociability)	↓ LPS-exposed males	Control males
	Delorme et al.^115^	Intra peritoneal injection of dams with Poly(I:C) (5 mg/kg; lot 1:086M4045V; Sigma-Aldrich) on E9.5 +Alternated between standard lighting for 4 weeks (LD1), constant lighting for 4 weeks (LL), then standard lighting for 4 weeks (LD2) (to test effects of disruption of circadian rhythm)	C57BL/6 J (Jackson Laboratories)	-10–12 weeks (last 12 days of LD1) -14–16 weeks (last 12 days of LL) -18–20 weeks (last 12 days of LD2)	Open field test	Horizontal activity (locomotor activity and reduced anxiety-like behavior)	↑ Poly(I:C)-exposed males	Control males
						Total distance covered (locomotor activity and reduced anxiety-like behavior)	↑ Poly(I:C)-exposed males	Control males
					Three-chamber social interaction test	Sociability proportion	↓ Poly(I:C)-exposed males	Control males
**Early Second**	Carpentier et al.^116^	Intra peritoneal injection of LPS (60 μg/kg at a concentration of 12 μg/mL in saline, *E. coli* [strain not specified], Sigma Aldrich) on E12.5 *All pups were fostered at birth to wild-type, naïve dams	C57BL/6 J (Jackson Laboratories)	4 weeks	3-chamber test with a stimulus mouse	Time spent interacting with novel stimulus mouse (sociability)	↓ LPS-exposed males	Control males
				5 weeks	Delayed match to place task	Time to find the hidden platform (spatial memory)	↑ LPS-exposed males	Control males
						Thigmotaxis (i.e., perimeter-circling; anxiety-like behavior)	↑ LPS-exposed males	Control males
						Number of times returning to previous day’s platform (learning)	↓ LPS-exposed males	Control males
						Swim speed (locomotion, motivation, and general health)	↓ LPS-exposed males	Control males
	Xuan et al.^119^	1) Intraperitoneal injection of dams with LPS (75 μg/kg, *E. coli* O127 B:8, Sigma Aldrich) on E11.5 and E12 or 2) Intraperitoneal injection of dams with Poly(I:C) potassium salt (20 mg/kg, Sigma Aldrich) in saline (4 mg/mL) on E12.5	C57BL/6 (laboratory of origin not specified)	7–8 weeks	Modified 3-chamber social test	Preference for the social chamber (sociability)	↓ LPS-exposed females	Control females
				9–10 weeks	Marble burying	Number of marbles buried (anxiety-like or obsessive-compulsive/repetitive behavior)	↑ LPS-exposed males	Control males
							↑ Poly(I:C)-exposed males	Control males
	Braun et al.^83^	Intraperitoneal injection of dams with LPS (30 μg/kg or 60 μg/kg, *E. coli* [strain not specified], Sigma Aldrich) on E12.5 *All pups were fostered at birth to wild-type, naïve dams	C57BL/6 J (Jackson Laboratories)	P21	Juvenile play paradigm	Initiation of play (social development)	↑ LPS-exposed females	Control females
					Open field test	Exploration of the center of the chamber over time (habituation)	↓ LPS-exposed females	Control females
					Modified 3-chamber social test	Time interacting with stimulus mouse (sociability)	↓ LPS-exposed males	Control males
				6–8 weeks	Delayed match to place water maze task	Time to find the platform (spatial memory)	↓ LPS-exposed males	LPS-exposed females
						Swim speed (locomotion, motivation, and general health)	↓ LPS-exposed males	LPS-exposed females
						Thigmotaxis (i.e., perimeter-circling; anxiety-like behavior)	↑ LPS-exposed males	LPS-exposed females
						Number of crossings over the location of the previous day’s platform (memory)	↓ LPS-exposed males	LPS-exposed females
						Number of entries into an annulus around the current platform (spatial memory)	↓ LPS-exposed males	LPS-exposed females
					Marble-burying task	Number of marbles buried (anxiety-like or obsessive-compulsive/repetitive behavior)	↑ LPS-exposed males	Control males
	Zhao et al.^117^	Intraperitoneal injection of dams with Poly(l:C) (20 mg/kg, tlrl-picw, lot number PIW-41–03, InvivoGen) on E12	C57BL/6 J mice from the Jackson Laboratories	P70	Social interaction test	Time spent interacting with novel mouse (sociability)	↓ Poly(I:C)-exposed males	Control males
						Time spent in interaction zone (sociability)	↓ Poly(I:C)-exposed male	Control males
**Late Second**	Bergeron et al.^118^	Intraperitoneal injection of inactivated serotype Ia *Streptococcus agalactiae* (group B streptococcus, GBS, 1×10^9^ CFU in 100 μL every 12 h from E19 until the end of gestation	Rats from Charles River Laboratories (strain not specified)	P9	Olfactory discrimination	Number of successes to reach a familiar odor (nest-seeking behavior)	↓ GBS-exposed males	Control males
						Latency to reach a familiar odor (nest-seeking behavior)	↑ GBS-exposed males	Control males
				P15-P25	Open field test	Total distance traveled (locomotor activity and reduced anxiety-like behavior)	↓ GBS-exposed males	Control males
						Duration of mobility (locomotor activity and reduced anxiety-like behavior)	↓ GBS-exposed males	Control males
				P40	Social interaction test	Total duration of social interactions (sociability)	↓ GBS-exposed males	Control males
							↑ GBS-exposed females	Control females
				P30-P35	Pre-pulse inhibition	Mean pre-pulse inhibition (sensorimotor gating)	↓ GBS-exposed males	Control males
				P30-P40	Rota rod	Latency to fall (motor function)	↓ GBS-exposed males	Control males
	Fernández de Cossío et al.^84^	Intraperitoneal injection of dams with LPS (100 μg/kg, *E. coli* 0111:B4, Sigma Aldrich) on E15	C57BL/6 mice from Charles River Laboratories	8 weeks	Marble burying	Number of marbles buried (anxiety-like or obsessive-compulsive/repetitive behavior)	↑ LPS-exposed females	Control females
				9 weeks	3-chamber test	Sociability index	↓ LPS-exposed males	LPS-exposed females
	Makinson et al.^85^	Intrauterine injection of dams with LPS (50 μg, *E*. *coli* 055: B5, Sigma Aldrich) on E15 via mini laparotomy under isoflurane anesthesia	CD-1 outbred mice from Charles River Laboratories	12–14 weeks	Elevated zero maze	Time spent in the open arms (reduced anxiety-like behavior)	↑ LPS-exposed offspring -No difference between sexes	Control offspring
						Number of transitions (reduced anxiety-like behavior)		
					Light-dark box	Time spent in the light (reduced anxiety-like behavior)		
						Number of transitions (exploratory behavior)		
					Open field	Activity in the peripheral zone (anxiety-like behavior)		
						Number of rearings (exploratory behavior)		
	Makinson et al.^88^	Intrauterine injection of dams with LPS (50 μg/100 μL, *E. coli* 055:B5, Sigma Aldrich) on E15 via mini laparotomy under isoflurane anesthesia	B62DF1/J hybrid dams bred from DBA/2 J males and C57BL/6 J females from The Jackson Laboratories	12–31 weeks	Fixed Ratio 1 test	Time to reach criterion (basic cognition)	↓ LPS-exposed females	Control females
					Progressive Ratio test	Breakpoint (motivation)	↑ LPS-exposed females	Control females
	Allard et al.^81^	Intraperitoneal injection of dams with live serotype la *Streptococcus agalactiae* (group B streptococcus, GBS, 10^8^-10^9^ CFU/100 μL saline) on E19	Lewis rats from Charles River Laboratories	P7	Ultrasonic vocalizations	Total number (communication)	↓ GBS-exposed males	Control males
							↓ GBS-exposed males	GBS-exposed females
						Total duration (communication)	↓ GBS-exposed males	Control males
							↓ GBS-exposed males	GBS-exposed females
				P9	Nest-seeking behavioral test	Latency to reach maternal odor (communication)	↑ GBS-exposed males	Control males
							↑ GBS-exposed males	GBS-exposed females
				P20	Open field test	Total distance traveled (locomotor activity and reduced anxiety-like behavior)	↑ GBS-exposed males	Control males
						Duration of mobility (locomotor activity and reduced anxiety-like behavior)	↑ GBS-exposed males	Control males
				P40	Social interactions test	Number of social interactions (sociability)	↓ GBS-exposed males	Control males
							↓ GBS-exposed males	GBS-exposed females
	Allard et al.^120^	Intraperitoneal injection of inactivated serotype Ia *Streptococcus agalactiae* (group B streptococcus, GBS) or inactivated serotype III GBS every 12 h from G19-G22	Lewis rats from Charles River Laboratories	P35 & P40	Rota rod test	Time before falling off rod (motor function)	↑ GBS-exposed males	GBS-exposed females
							↓ GBS-exposed females	Control females
						Speed before falling off rod (motor function)	↓ GBS-exposed females	Control females
				P105-P110	Elevated plus maze	Distance traveled in the open arms (reduced anxiety-like behavior)	↓ GBS-exposed males	GBS-exposed females
						Number of entries into open arms (reduced anxiety-like behavior)	↓ GBS-exposed males	GBS-exposed females
							↑ GBS-exposed females	Control females
						Total distance traveled (locomotion)	↓ GBS-exposed males	GBS-exposed females
							↑ GBS-exposed females	Control females
	Allard et al.^121^	Intraperitoneal injection of inactivated serotype Ia *Streptococcus agalactiae* (group B streptococcus, GBS) or inactivated serotype III GBS every 12 h from G19-G22	Lewis rats from Charles River Laboratories	P25	Open field test	Total distance traveled (locomotor activity and reduced anxiety-like behavior)	↓ GBS-exposed males	Control males
							↓ GBS-exposed males	GBS-exposed females
						Duration of mobility (locomotor activity and reduced anxiety-like behavior)	↓ GBS-exposed males	Control males
						Number of line crosses (locomotor activity and reduced anxiety-like behavior)	↓ GBS-exposed males	Control males
	Zhang et al.^131^	Intraperitoneal injection of dams with LPS (50 μg/kg, Sigma Aldrich, strain not specified) at E15-E17	CD-1 mice from the Animal Experimental Center of Anhui Medical University	3 months or 13 months	Morris water maze	Escape latency (spatial memory)	↑ LPS-exposed males at 3 and 13 months	Control males

*GBS* group B streptococcus, *LPS* lipopolysaccharide, *Poly(I:C)* polyinosinic:polycytidylic acid.

**Table 4.** Alterations in cerebral structure following murine maternal immune activation.

Trimester Equivalent	Study	Study model	Mouse or rat strain	Age at outcome	Method	Measurement	Findings	Comparison Group
**First**	Harvey and Boksa^122^	1) Subcutaneous injection of dams with LPS (0.2 mg/kg, *E*. *coli* 0111:B4, Sigma Aldrich) on E9 or 2) Tail vein injection of dams with Poly(I:C) (20 mg/kg, Sigma Aldrich) on E8.5	Outbred Swiss-Webster mice (Charles River Laboratories)	P14	Neuronal cell density of the SO of the CA1 subfield of the hippocampus (via immunohistochemistry)	Neuronal density in the SO of the ventral hippocampus measured by NeuN+ staining	↑ LPS-exposed offspring -No difference between sexes	Control offspring
							↑ LPS-exposed offspring -No difference between sexes	Poly (I:C-exposed offspring
	Solmaz et al.^114^	Intraperitoneal injection of dams with LPS (100 μg/kg, *E. coli* O127:B8) on E10	Sprague-Dawley rats from the Experimental Animal Laboratory of Bilim University	P50	Histopathology of hippocampal CA1 regions	Total neuron count in the hippocampal CA1 region	↓ LPS-exposed males	Control males
**Early Second**	Braun et al.^83^	Intraperitoneal injection of dams with LPS (30 μg/kg or 60 μg/kg, *E.coli* [strain not specified], Sigma Aldrich) on E12.5 *All pups were fostered at birth to wild-type, naïve dams	C57BL/6 J (Jackson Laboratories)	P42-P56	Immunohistochemistry (Satb2 and parvalbumin staining) of the anterior cortex	Parvalbumin+ (inhibitory) neuronal density	↓ LPS-exposed males	Control males
						Satb2 +/DAPI per mm^2^	↓ LPS-exposed males	Control males
					Immunohistochemistry (Satb2 and parvalbumin staining) of the posterior cortex	Satb2+ density	↓ LPS-exposed males	Control males
						Parvalbumin+ density	↓ LPS-exposed males	Control males
							↑ LPS-exposed females	Control females
						DAPI+ density	↑ LPS-exposed males	Control males
						PV/Satb2 ratio	↑ LPS-exposed males	Control males
**Late Second**	Bergeron et al.^118^	Intraperitoneal injection of inactivated serotype la *Streptococcus agalactiae* (group B streptococcus, GBS) 1×10^9^ CFU in 100 μL every 12 h from E19 until the end of gestation	Rats from Charles River Laboratories (strain not specified)	P40	Histology and immunohistochemistry of forebrains	Lateral ventricle area	↑ GBS-exposed males	Control males
						MBP staining	↓ GBS-exposed females	Control females
					Ultrastructure of the external capsule (via electron microscopy)	Patches of axonal loss	↑ GBS-exposed males	Control males
	Dada et al.^123^	Intrauterine injection of dams with LPS (50 μg, *E coli*, 055:B5, Sigma Aldrich) on E17 via mini laparotomy under isoflurane anesthesia	CD-1 outbred mice from Charles River Laboratories	P60	Hippocampal volume (via Magnetic Resonance Imaging)	Hippocampal volume	↓ LPS-exposed offspring -No difference between sexes	Control offspring
	Fernández de Cossío et al.^84^	Intraperitoneal injection of dams with LPS (100 μg/kg, *E. coli* 0111:B4, Sigma Aldrich) on E15	C57BL/6 mice from Charles River Laboratories	P15	Golgi-Cox staining and neuron tracing of the hippocampus	Spine density of the granule cells of the hippocampus	↑ LPS-exposed males	Control males
	Makinson et al.^85^	Intrauterine injection of dams with LPS (50 μg, *E. coli* 055: B5, Sigma Aldrich) on E15 via mini laparotomy under isoflurane anesthesia	CD-1 outbred mice from Charles River Laboratories	PI 96	Immunohistochemistry	MBP in the corpus callosum	↓ LPS-exposed females	LPS-exposed males
	Allard et al.^81^	Intraperitoneal injection of dams with live serotype la *Streptococcus agalactiae* (group B streptococcus, GBS) 10^8^-10^9^ CFU/100 μL saline on E19	Lewis rats from Charles River Laboratories	P40	Histology of the forebrains	Corpus callosum thickness	↓ GBS-exposed males	Control males
						External capsule thickness	↓ GBS-exposed males	Control males
	Allard et al.^121^	Intraperitoneal injection of inactivated serotype la *Streptococcus agalactiae* (group B streptococcus, GBS) or inactivated serotype III GBS every 12 h from E19-E22	Lewis rats from Charles River Laboratories	P40	Immunohistochemistry analysis of primary motor cortices and corpus callosum of forebrains	Cortical thickness	↓ GBS-exposed males	Control males

*CA1* cornu ammonis 1, *DAPI* 4′,6-diamidino-2-phenylindole, *GBS* group B streptococcus, *LPS* lipopolysaccharide, *MBP* myelin basic protein, *Poly(I:C)* polyinosinic:polycytidylic acid, *SO* stratum oriens.

**Table 5.** Alterations in neuroimmunity following murine maternal immune activation.

Trimester equivalent	Study	Study model	Mouse strain	Age at outcome	Method	Measurement	Findings	Comparison Group
**First**	Solmaz et al.^114^	Intraperitoneal injection of dams with LPS (100 μg/kg, *E. coli* O127:B8) on E10	Sprague-Dawley rats from the Experimental Animal Laboratory of Bilim University	P50	Level of nerve growth factor in the brain (via ELISA)	Nerve growth factor	↓ LPS-exposed males	Control males
	Dijkstra et al.^124^	Intraperitoneal injection of dams with LPS (25 μg/kg in 100 μL sterile saline, *E. coli* 0111: B4, Sigma Aldrich) on E10.5* *Failed adenovirus (1 × 10^9^ sFlt-1 in 100 μL PBS) injection retro-orbitally on E8.5 (= failed induction of preeclampsia) +added a western-style diet (WSD) vs control diet (CTRD) variable beginning at 12 weeks of age	C57BL/6 J (Charles River Laboratories)	24 weeks	Cortical expression of genes involved in neuroimmune homeostasis and activation	*P2ry12*	↓ LPS-exposed CTRD males	Control CTRD males
							↓ LPS-exposed WSD females	Control WSD females
						*Tmem119*	↓ LPS-exposed CTRD males	Control CTRD males
							↓ LPS-exposed WSD females	Control WSD females
						*Tyrobp*	↓ LPS-exposed CTRD males	Control CTRD males
						*Ctsd*	↓ LPS-exposed CTRD males	Control CTRD males
						*Slc1a2*	↑ LPS-exposed CTRD females	Control CTRD females
**Late Second**	Bergeron et al.^118^	Intraperitoneal injection of inactivated serotype la *Streptococcus agalactiae* (group B streptococcus, GBS) 1 × 10^9^ CFU in 100 μL every 12 h from E19 until the end of gestation	Rats from Charles River Laboratories (strain not specified)	P40	Immunohistochemistry of external capsule	Iba-1+ cells (microglia/macrophages)	↓ GBS-exposed females	Control females
	Dada et al.^123^	Intrauterine injection of dams with LPS (50 μg, *E coli*, 055:B5, Sigma Aldrich) on E17 via mini laparotomy under isoflurane anesthesia	CD-1 outbred mice from Charles River Laboratories	P120	Frequency of macrophages (via flow cytometry)	Macrophage infiltration of the cortex	↑ LPS-exposed males	Control males
	Fernández de Cossiío et al.^84^	Intraperitoneal injection of dams with LPS (100 μg/kg, *E. coli* 0111:B4, Sigma Aldrich) on E15	C57BL/6 mice from Charles River Laboratories	P15	RT-qPCR of molecular pruning signals in the hippocampus	*Cx3cr1*	↓ LPS-exposed males	Control males
						*C3*	*i* LPS-exposed males	LPS-exposed females
	Makinson et al.^85^	Intrauterine injection of dams with LPS (50 μg, *E. coli* 055: B5, Sigma Aldrich) on E15 via mini laparotomy under isoflurane anesthesia	CD-1 outbred mice from Charles River Laboratories	50 weeks, 2 h after intraperitoneal injection of saline or LPS (10 μg)	Gene expression profiling within the hypothalamus, amygdala, and prefrontal cortex (via RT-qPCR)	Hypothalamic *Il1b*	↑ LPS-exposed males receiving LPS	Control males receiving LPS
						Hypothalamic *Cxcl10*	↑ LPS-exposed males receiving LPS	Control males receiving LPS
						Amygdala *Il1b*	↑ LPS-exposed males receiving LPS	Control males receiving LPS
						Amygdala *Cxcl10*	↑ LPS-exposed males receiving LPS	Control males receiving LPS
						Amygdala *Tnf*	↑ LPS-exposed males receiving LPS	Control males receiving LPS
						Amygdala *Socs3*	↑ LPS-exposed males receiving LPS	Control males receiving LPS
						Prefrontal cortex *Il1b*	↑ LPS-exposed males receiving LPS	Control males receiving LPS
						Prefrontal cortex *Cxcl10*	↑ LPS-exposed males receiving LPS	Control males receiving LPS
						Prefrontal cortex *Tnf*	↑ LPS-exposed males receiving LPS	Control males receiving LPS
	Makinson et al.^88^	Intrauterine injection of dams with LPS (50 μg/100 μL, *E. coli* 055:B5, Sigma Aldrich) on E15 via mini laparotomy under isoflurane anesthesia	B62DF1/J hybrid dams bred from DBA/2 J males and C57BL/6 J females from The Jackson Laboratories	12–31 weeks, 24 h after intraperitoneal injection of LPS (10 μg)	Gene expression profiling of 31 genes within the prefrontal cortex and hypothalamus (via RT-qPCR)	Prefrontal cortex *Cr3*	↑ LPS-exposed females	LPS-exposed males
						Prefrontal cortex *Csf1r*	↑ LPS-exposed females	LPS-exposed males
						Prefrontal cortex *Cx3cr1*	↑ LPS-exposed females	LPS-exposed males
						Hypothalamic *Nr3c1*	↑ LPS-exposed females	LPS-exposed males
	Allard et al.^121^	Intraperitoneal injection of inactivated serotype la *Streptococcus agalactiae* (group B streptococcus, GBS) or inactivated serotype III GBS every 12 h from E19-E22	Lewis rats from Charles River Laboratories	P40	Immunofluorescence analyses of primary motor cortices and corpus callosum of forebrains	Iba-1+ cells (microglia/macrophages)	↓ GBS-exposed males	Control males

*ELISA* enzyme-linked immunosorbent assay, *GBS* group B streptococcus, *LPS* lipopolysaccharide, *PBS* phosphate buffered saline, *RT-qPCR* reverse transcription quantitative polymerase chain reaction.

**Table 6.** Alterations in brain gene, protein, and metabolite expression following murine maternal immune activation.

Trimester equivalent	Study	Study model	Mouse or rat strain	Age at outcome	Method	Measurement	Findings	Comparison Group
**First**	Harvey and Boksa^122^	1) Subcutaneous injection of dams with LPS (0.2 mg/kg, *E. coli* 0111:B4, Sigma Aldrich) on E9 or 2) Tail vein injection of dams with Poly(I:C) (20 mg/kg, Sigma Aldrich) on E8.5	Outbred Swiss-Webster mice (Charles River Laboratories)	P14	Reelin+ cell density of the SO of the CA1 subfield of the hippocampus (via immunohistochemistry)	Reelin+ cells in the ventral SO	↑ LPS-exposed males	LPS-exposed females
							↑ Poly(I:C)-exposed males	Poly(I:C)-exposed females
				P28	GAD67+ and reelin+ cell density of the SO of the CA1 subfield of the hippocampus (via immunohistochemistry)	GAD67+ cell density in the ventral SO	↑ LPS-exposed males	Control males
							↑ LPS-exposed males	Poly(I:C)-exposed males
							↑ Poly(I:C)-exposed females	Control females
							↑ Poly(I:C)-exposed females	LPS-exposed females
						GAD67+ cell density in the dorsal SO	↓ LPS-exposed males	LPS-exposed females
							↓ Poly(I:C)-exposed males	Poly(I:C)-exposed females
						Reelin+ cells in the ventral SO	↓ LPS-exposed males	LPS-exposed females
							↓ Poly(I:C)-exposed males	Poly(I:C)-exposed females
						Reelin+ cells in the dorsal SO	↓ Poly(I:C)-exposed males	Control males
							↓ Poly(I:C)-exposed females	Control females
	Connors et al.^113^	Intraperitoneal injection of dams with LPS (100 μg/kg, *E. coli* 026:B6) on E11	Sprague-Dawley rats from Charles River Laboratories	P42	Hippocampal levels of glutamate (via LC-MS)	Glutamate	↓ LPS-exposed males	Control males
					Hippocampal levels of corticosterone (via LC-MS) and the glucocorticoid receptor (via western blot)	Corticosterone	↑ LPS-exposed males	Control males
							↑ LPS-exposed males	LPS-exposed females
						Glucocorticoid receptor/actin ratio	↓ LPS-exposed males	Control males
	Solmaz et al.^114^	Intraperitoneal injection of dams with LPS (100 μg/kg, *E. coli* O127:B8) on E10	Sprague-Dawley rats from the Experimental Animal Laboratory of Bilim University	P50	GAD-67 and 5-HIAA levels in the brain (via ELISA)	GAD-67	↓ LPS-exposed offspring -No difference between sexes	Control offspring
						5-HIAA	↓ LPS-exposed offspring -No difference between sexes	Control offspring
	Zhao et al.^117^	Intraperitoneal injection of dams with Poly(I:C) (20 mg/kg, tlrl-picw, lot number PIW-41–03, InvivoGen) on E12	C57BL/6 J mice from the Jackson Laboratories	P85	Ventral hippocampal expression of genes involved in HPA axis (via RT-qPCR)	*Oxtr*	↑ Poly(I:C)-exposed males	Control males
							↓ Poly(I:C)-exposed females	Control females
						*Crh*	↑ Poly(I:C)-exposed males	Control males
						*Crhr1*	↑ Poly(I:C)-exposed males	Control males
						*Nr3c1*	↑ Poly(I:C)-exposed males	Control males
**Late Second**	Makinson et al.^85^	Intrauterine injection of dams with LPS (50 μg*, E. coli* 055: B5, Sigma Aldrich) on E15 via mini laparotomy under isoflurane anesthesia	CD-1 outbred mice from Charles River Laboratories	50 weeks	*Gadi* mRNA levels in the hypothalamus and *Drd2* mRNA levels in the amygdala (via RT-qPCR)	Hypothalamic *Gad1*	↑ LPS-exposed males	Control males
						Amygdala *Drd2*	↑ LPS-exposed males	Control males
	Zhanq et al.^131^	Intraperitoneal injection of dams with LPS (50 μg/kg, Sigma Aldrich, strain not specified) at E15-E17	CD-1 mice from the Animal Experimental Center of Anhui Medical University	3 months	Immunohistochemistry and western blot of H3K9me3 and H4K12ac in the hippocampus	Total hippocampal levels of H3K9me3	↑ LPS-exposed males	Control males
							↑ LPS-exposed males	LPS-exposed females
						Total hippocampal levels of H4K12ac	↓ LPS-exposed males	Control males
				13 months	Immunohistochemistry and western blot of H3K9me3 in the hippocampus	Total hippocampal levels of H3K9me3	↑ LPS-exposed males	LPS-exposed females

*CA1* cornu ammonis 1, *ELISA* enzyme-linked immunosorbent assay, *HPA* hypothalamic-pituitary-adrenal, *LC-MS* liquid chromatography-mass spectrometry, *LPS* lipopolysaccharide, *mRNA* messenger ribonucleic acid, *Poly(I:C)* polyinosinic:polycytidylic acid, *RT-qPCR* reverse transcription quantitative polymerase chain reaction, *SO* stratum oriens.

**Table 7.** Metabolic outcomes following murine maternal immune activation.

Trimester equivalent	Study	Study model	Mouse or rat strain	Age at outcome	Measurement	Measurement	Findings	Comparison Group
**Late Second**	Zhao et al.^152^	Intraperitoneal injection of dams with LPS (50 μg/kg, strain not specified, Sigma Aldrich) daily from E15-E17	ICR (CD-1) outbred mice from Beijing Vital River (foundation colonies from Charles River Laboratories)	PI 20	Weight and fat coefficient	Weight	↓ LPS-exposed females	Control females
						Visceral fat tissue weight	↑ LPS-exposed males	Control males
						Fat coefficient ([the weight of adipose tissue/body weight] × 100%)	↑ LPS-exposed males	Control males
					Biochemical parameters	Serum leptin levels (via ELISA)	↑ LPS-exposed males	Control males
					Glucose tolerance test	Glucose levels (via glucometer)	↑ LPS-exposed males	Control males
						Area under the curve for the two-hour time course	↑ LPS-exposed males	Control males
					Insulin tolerance test	Remaining percentage of glucose following insulin administration (via glucometer)	↑ LPS-exposed males	Control males
						Area under the curve for the two-hour time course	↑ LPS-exposed males	Control males
	Chin et al.^86^	Intraperitoneal injection of dams with LPS (20 μg/kg. *Salmonella typhimurium*, Sigma Aldrich) on E16 ± intraperitoneal injection of dams with (+)-naloxone (60 mg/kg in 100 μL PBS with 0.1% BSA) or vehicle within 5 min of LPS injection, then additional injections at 12-hour intervals on E17, E17.5, and E18	C57BL/6 J mice from the Jackson Laboratories	20 weeks	Body morphometry analysis	Quadriceps muscle weight	↓ LPS-exposed males	Control males
						Epidydimal fat	↑ LPS-exposed males	Control males
						Muscle: central fat ratio	↓ LPS-exposed males	Control males
					Plasma leptin and adiponectin levels (via Luminex multiplex assay)	Plasma adiponectin levels	↓ LPS-exposed females	Control females
						Leptin: adiponectin ratio	↑ LPS-exposed females	Control females
	Dijkstra et al.^124^	Intraperitoneal injection of dams with LPS (25 μg/kg in 100 μL sterile saline, *E. coli* 0111: B4, Sigma Aldrich) on E10.5* *Failed adenovirus (1×10^9^ sFlt-1 in 100 μL PBS) injection retro-orbitally on E8.5 (= failed induction of preeclampsia) +added a western-style diet (WSD) vs control diet (CTRD) variable beginning at 12 weeks of age	C57BL/6 J mice from Charles River Laboratories	Week 12	Fat mass	Fat mass	↑ LPS-exposed females	Control females
				Week 24	Fat mass and food intake	Fat mass	↑ LPS-exposed males on CTRD	Control males on CTRD
							↑ LPS-exposed females on WSD	Control females on WSD
						Daily food intake	↑ LPS-exposed females on WSD	Control females on WSD
						Gonadal white adipose tissue weight	↑ LPS-exposed females on WSD	Control females on WSD
						Inguinal white adipose tissue weight	↑ LPS-exposed females on WSD	Control females on WSD
					Liver and gonadal white adipose tissue expression of genes involved in fatty acid metabolism (via RT-qPCR)	Hepatic expression of *Fasn*	↑ LPS-exposed males on CTRD	Control males on CTRD
						Gonadal adipose tissue expression of *Leptin*	↑ LPS-exposed males on CTRD	Control males on CTRD
							↑ LPS-exposed females on WSD	Control females on WSD
	Ushida et al.^148^	Intraperitoneal injection of dams with LPS (10 μg/kg; *E. coli* 0111:B4; Sigma-Aldrich) on E13.5 and then daily from E14.5–16.5 (40 μg/kg/day)	Wistar rats from Charles River Laboratories	4–24 weeks	Weight	Post-weaning weight	↓ LPS-exposed females	Control females
				24 weeks	Visceral fat accumulation	Mesenteric fat	↑ LPS-exposed males	Control males
						Epididymal/parametrial fat	↑ LPS-exposed males	Control males
						Retroperitoneal fat	↑ LPS-exposed males	Control males

*BSA* bovine serum albumin, *ELISA* enzyme-linked immunosorbent assay, *LPS* lipopolysaccharide, *PBS* phosphate buffered saline, *RT-qPCR* reverse transcription quantitative polymerase chain reaction.

**Table 8.** Miscellaneous systemic outcomes following murine maternal immune activation.

Trimester equivalent	Study	Study model	Rat strain	Age at outcome	Method	Measurement	Findings	Comparison Group
**Early Second**	Ushida et al.^148^	Intraperitoneal injection of dams with LPS (10 μg/kg; *E*. *coli* 0111:B4; Sigma-Aldrich) on E13.5 and then daily from E14.5–16.5 (40 μg/kg/day)	Wistar rats from Charles River Laboratories	24 weeks	CBC (using an ABC Vet Animal Blood Counter)	Red blood cell number	↓ LPS-exposed females	Control females
						Hematocrit	↓ LPS-exposed females	Control females
					Coagulation function (via thromboelastography)	Clot formation time	↑ LPS-exposed females	Control females
				E17.5 -Female offspring of dams previously treated with LPS or saline during mid-gestation were bred at 10–14 weeks of age with non-experimental males	F1 pregnancy uteroplacental immunohistochemistry	Glut1 staining in the mesometrial triangle and the labyrinth	↓ Female dams born to LPS-exposed mothers	Female dams born to control mothers
						CD68+ staining in the mesometrial triangle, junctional zone, and the labyrinth	↑ Female dams born to LPS-exposed mothers	Female dams born to control mothers
					F1 pregnancy fetal growth restriction rate	Fetal growth restriction rate	↑ Female dams born to LPS-exposed mothers	Female dams born to control mothers

*F1* first filial generation, *LPS* lipopolysaccharide, *RT-qPCR* reverse transcription quantitative polymerase chain reaction.

## Data Availability

Data sharing is not applicable to this article as no datasets were generated or analyzed during the current study.

## IMPACT:

- Few investigations of chorioamnionitis-related outcomes stratify analyses by fetal sex, leaving key biologic differences unexplored.
- Male and female fetuses mount distinct placental and systemic inflammatory responses to chorioamnionitis, which likely contribute to differential neurodevelopmental, pulmonary, metabolic, and cardiac outcomes.
- This review provides the first comprehensive synthesis of sex-specific responses to chorioamnionitis and highlights the need to analyze sex as a biological variable in future outcome-related studies.
